# Supplementary material for: Interaction of Glia Cells with Glioblastoma and Melanoma Cells under the Influence of Phytocannabinoids
Source: Cells. 2022 Jan 3;11(1):147. doi: 10.3390/cells11010147 (PMC8750637; doi:10.3390/cells11010147)
Supplement: Supplementary file 1 [file cells-11-00147-s001.zip › cells-1488917-supplementary.pdf]

Supplementary Materials:

Table S1. Sample size.

**Experiment / Treatment**	**LN229**	**A375**
Single Cell Migration	93	143
CTL		
CBD	58	76
THC	60	56
THC+CBD	112	64
ACM	106	135
ACM+CBD	112	67
ACM+THC	87	97
ACM+THC+CBD	88	51
BV2CM	83	81
BV2CM+CBD	42	88
BV2CM+THC	42	75
BV2+THC+CBD	64	67
Collective migration		
CTL	24	28
CBD	25	32
THC	25	32
THC+CBD	26	35
ACM	21	37
ACM+CBD	19	38
ACM+THC	22	38
ACM+THC+CBD	20	31
BV2CM	23	39
BV2CM+CBD	20	41
BV2CM+THC	23	34
BV2+THC+CBD	23	39
Aggregation	74	48
CTL	73	46
CBD	78	45
THC	69	33
THC+CBD	39	20
30% A	19	10
A+CBD	16	9
A+THC	42	13
A+THC+CBD	30	29
10% MG	14	9
MG+CBD	22	12
MG+THC	12	4
MG+THC+CBD		
Ratio establishment		
5%MG	23	23
10%MG	19	20
15%MG	14	19
30%MG	10	16

5%A	11	15
10%A	14	14
15%A	14	12
30%A	6	8

Abbreviations: CTL: control, CBD: cannabidiol, THC: tetrahydrocannabinol, ACM: astrocyte conditioned media, BV2CM: BV2 cell conditioned media, A: astrocytes, MG: microglia, x%: relative proportion of MG or A to total cell number.

Table S2. Characteristic spheroid size, aggregation time and aggregation speed.

	**LN229**			**A375**		
	$A_0/10^5$ in px	t_0 in h	$A_0/10^5/t_0$ in px/h	$A_0/10^5$ in px	t_0 in h	$A_0/10^5/t_0$ in px/h
CTL	1.15	4.4	0.261	2.68	17.6	0.152
CBD	1.92	4.5	0.426	3.08	30.2	0.102
THC	2.07	4.1	0.505	2.62	17.1	0.153
THC+CBD	1.98	4.8	0.412	2.61	21.8	0.120
30% A	0.91	4.7	0.194	2.75	17.1	0.161
A+CBD	0.95	6.5	0.146	2.50	20.0	0.125
A+THC	1.08	4.6	0.235	3.62	18.8	0.193
A+THC+CBD	1.00	6.0	0.167	2.69	17.5	0.154
10% MG	1.33	3.7	0.359	3.03	43.1	0.07
MG+CBD	1.27	3.7	0.343	1.96	24.5	0.08
MG+THC	1.13	4.0	0.283	2.05	31.7	0.06
MG+THC+CBD	0.69	4.5	0.153	1.98	20.4	0.10

Abbreviations: CTL: control, CBD: cannabidiol, THC: tetrahydrocannabinol, A: astrocytes, MG: microglia, x%: relative proportion of MG or A to total cell number.

Table S3. Proliferation index of cells inside of spheroids.

	**LN229**			**A375**		
**Treatment**	**Mean**	**Standard deviation**	**Sample Size**	**Mean**	**Standard deviation**	**Sample Size**
CTL	0.027	0.017	6	0.005	0.005	9
CBD	0.027	0.031	15	0.004	0.005	4
THC	0.026	0.013	6	0.042	0.024	3
THC+CBD	0.048	0.013	15	0.025	0.021	9
30% A	0.043	0.029	9	0.006	0.011	6
A+CBD	0.053	0.021	9	0.007	0.005	3
A+THC	0.034	0.019	10	0.018	0.020	15
A+THC+CBD	0.037	0.016	9	0.011	0.007	5
10% MG	0.029	0.010	3	0.015	0.017	10
MG+CBD	0.023	0.019	22	0.007	0.010	12
MG+THC	0.030	0.023	15	0.006	0.009	14
MG+THC+CBD	0.012	0.015	10	0.012	0.015	13

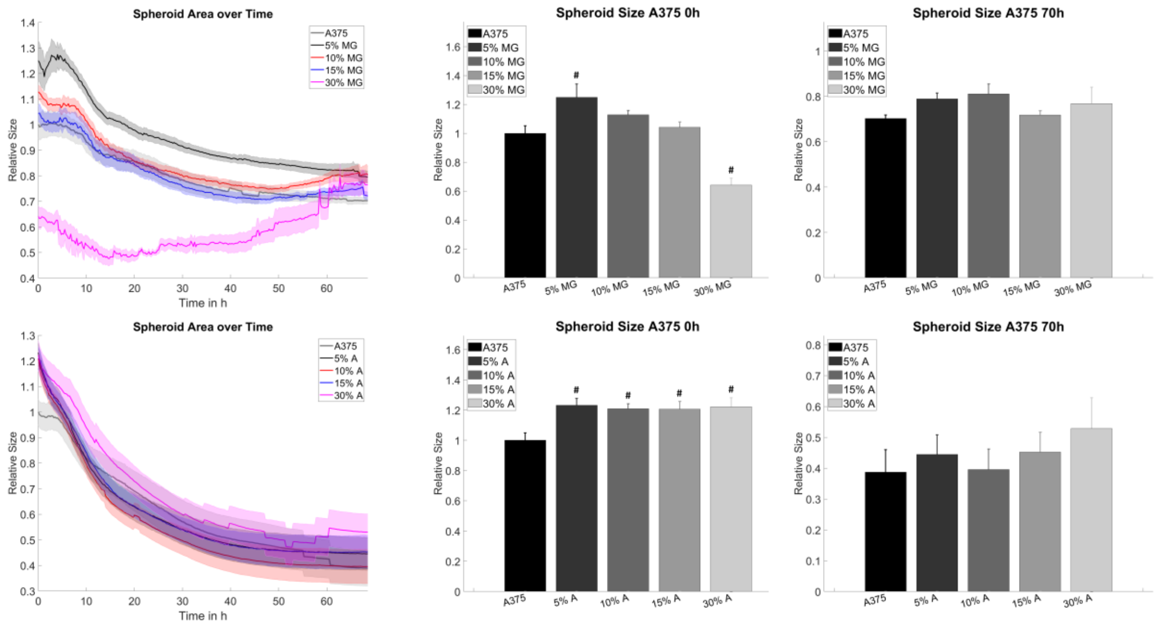

Figure S1: Establishment of astrocyte and microglia concentration for co-culture with A375 cells. Hashes (#) depict significant results against the untreated control. Errorbars and shaded areas depict the standard error of the mean. Abbreviations: CTL: control, CBD: cannabidiol, THC: tetrahydrocannabinol, A: astrocytes, MG: microglia, x%: relative proportion of MG or A to total cell number.

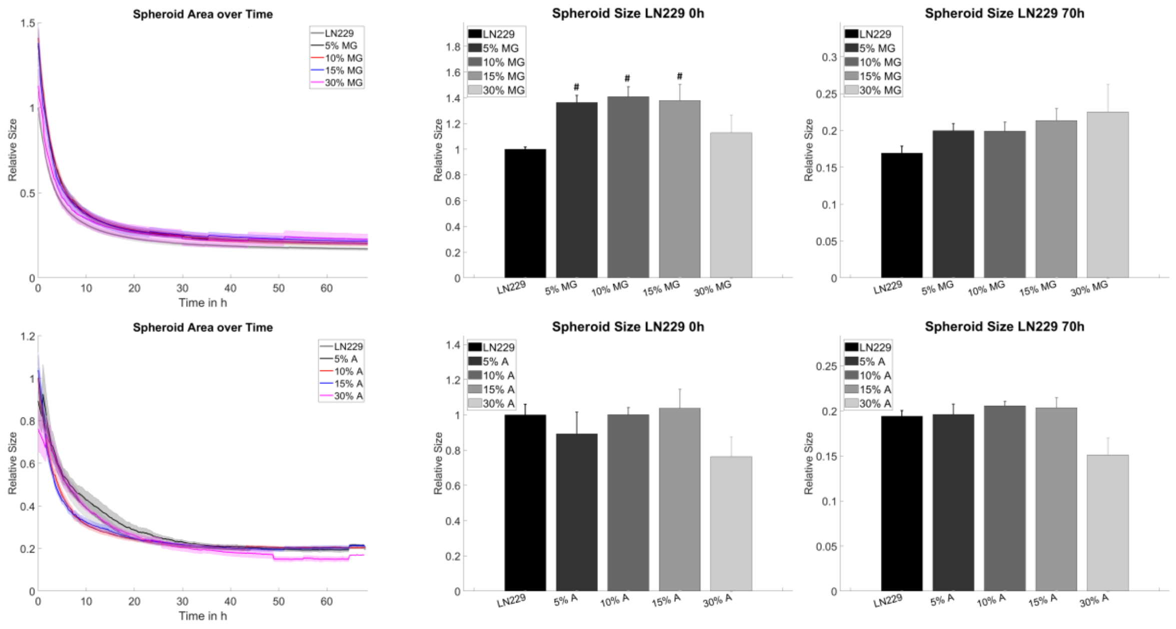

Figure S2: Establishment of astrocyte and microglia concentration for co-culture with LN229 cells. Hashes (#) depict significant results against the untreated control. Errorbars and shaded areas depict the standard error of the mean. Abbreviations: CTL: control, CBD: cannabidiol, THC: tetrahydrocannabinol, A: astrocytes, MG: microglia, x%: relative proportion of MG or A to total cell number.

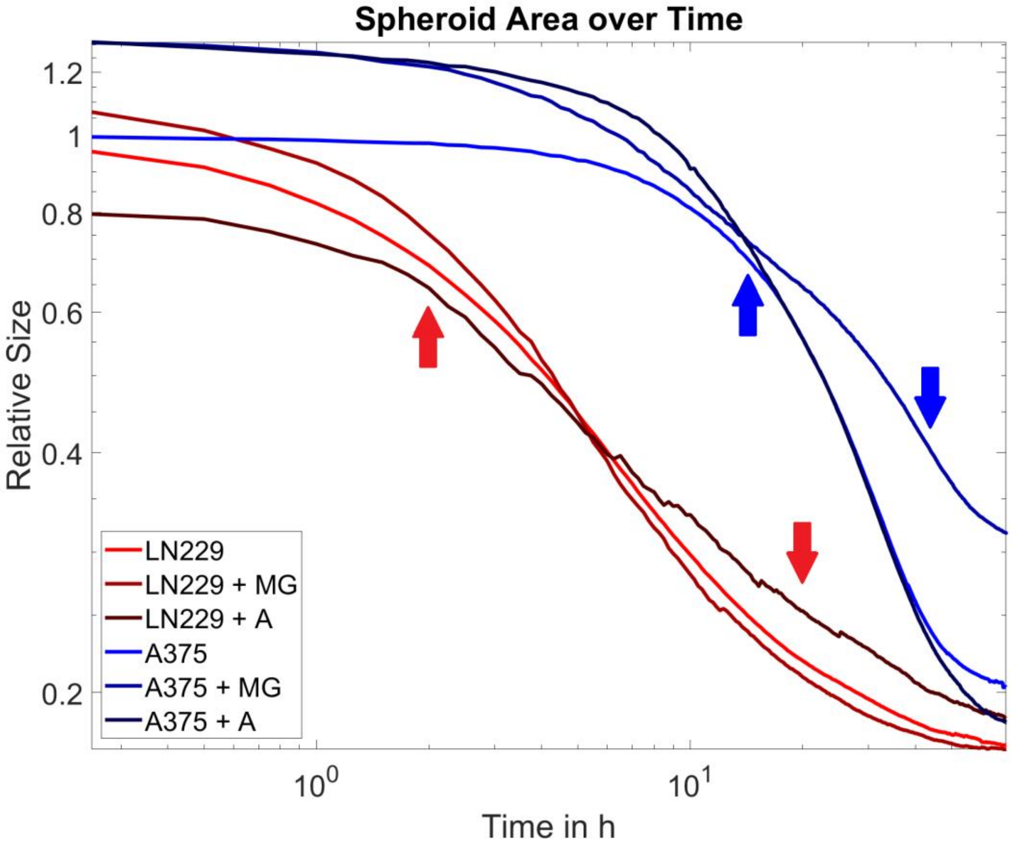

Figure S3: Spheroid aggregation dynamics. Log-Log-plot of the relative spheroid size as a function of time. The arrows indicate the start and end time used for fitting the exponential decay, to characterize spheroid aggregation speed. For LN229 cells it corresponds to 2-20 h and for A375 cells to 15-45 h. Abbreviations: A: astrocytes, MG: microglia.

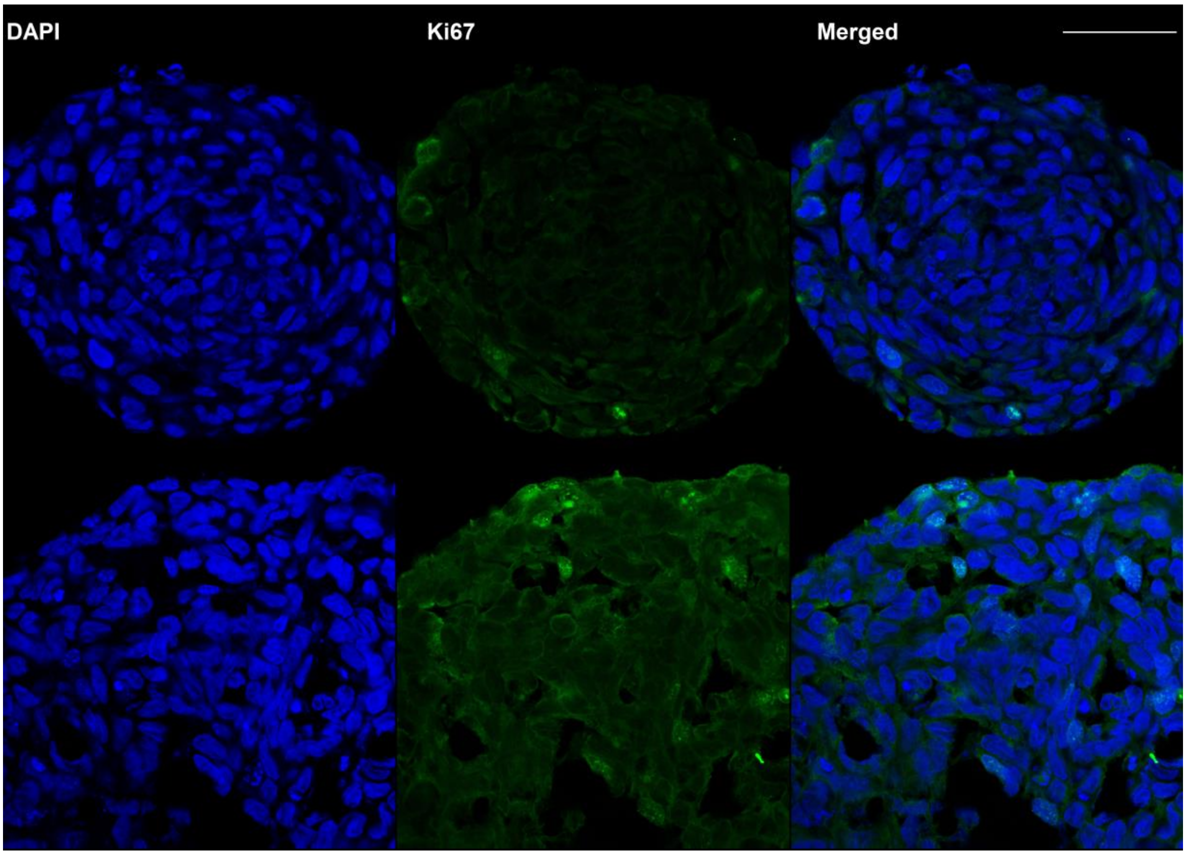

Figure S4: Representative labelings of sectioned spheroids for analysis of proliferation. For both image series DAPI is labeled in blue and the proliferation marker Ki67 is labeled in green. Please denote the very low number of Ki67 positive nuclei. The scale bar depicts 50 µm.

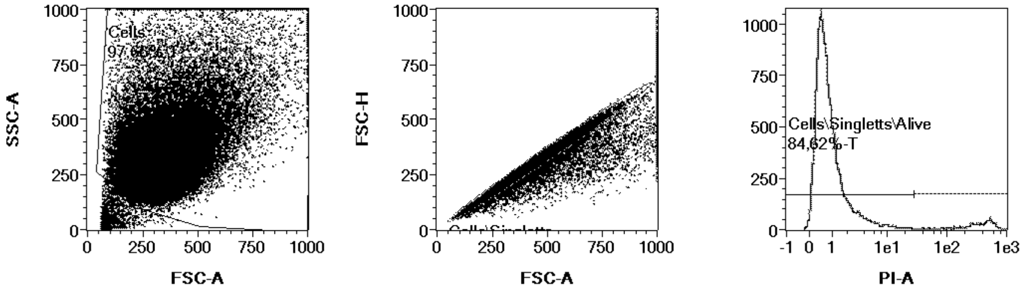

(a) A375 and astrocytes

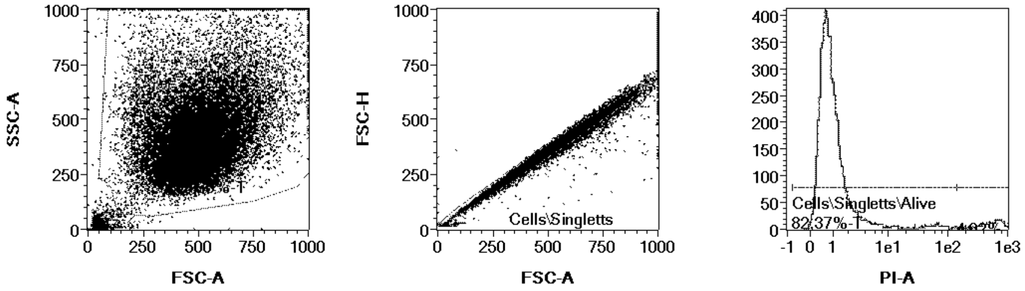

(b) A375 and microglia

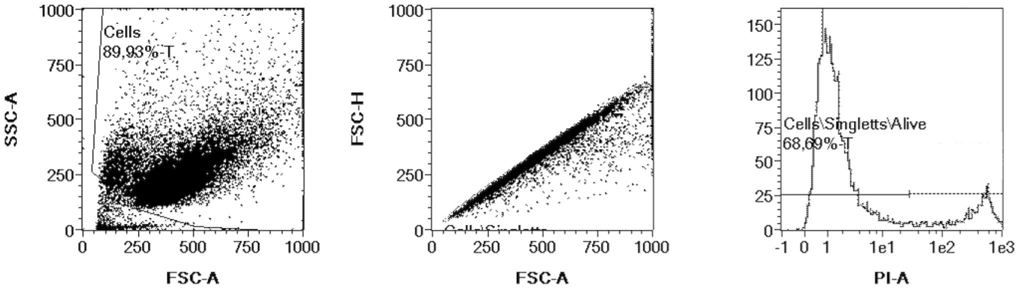

(c) LN229 and astrocytes

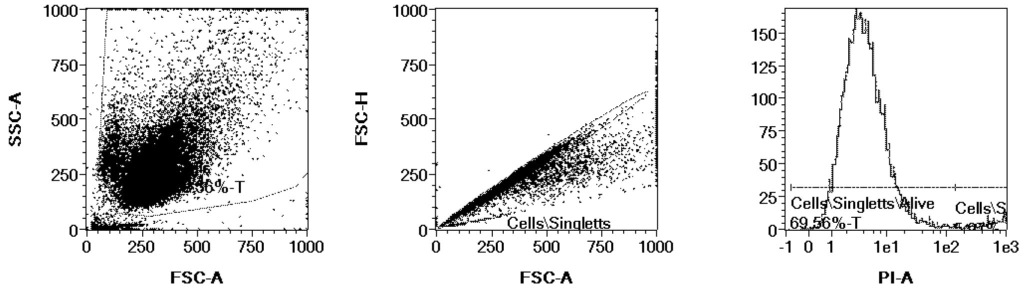

(d) LN229 and microglia

Figure S5: Gating strategy. This figure illustrates the gating strategy for identification of cells (first row), doublets (second row), and propidium iodide positive and negative cells (third row), for A375 cells co-cultured with astrocytes (a) or microglia (b) and LN229 cells co-cultured with astrocytes (c) and microglia (d).
